# Influence of Occupation on the Prevalence of Spinal Pain among Physiotherapists and Nurses

**DOI:** 10.3390/jcm11195600

**Published:** 2022-09-23

**Authors:** Aleksandra Bryndal, Sebastian Glowinski, Agnieszka Grochulska

**Affiliations:** 1 Department of Physiotherapy, Institute of Health Sciences, Slupsk Pomeranian University, Westerplatte 64, 76-200 Slupsk, Poland; 2 Institute of Physical Culture and Health, State Higher School of Vocational Education in Koszalin, Lesna 1, 75-582 Koszalin, Poland

**Keywords:** physiotherapists, nurses, neck pain, low back pain, medical staff, occupation

## Abstract

(1) Background: Neck pain (NP) and low back pain (LBP) are common musculoskeletal disorders, one of the major causes of disability globally. The aim of the study was to determine the influence of medical occupation (physiotherapist and nurse) on the prevalence of spinal pain, functional status and degree of disability. (2) Methods: a total of 544 people (462 females (84.9%) and 82 males (15.1%)), licensed to practice as a physiotherapist (n1 = 240 (44.1%)) or nurse (n2 = 304 (55.9%)) in Poland completed a special questionnaire designed by the authors of the study, and were assessed using the Neck Disability Index (NDI, Polish language version) and Revised Oswestry Disability Index (ODI, Polish language version). (3) Results: Compared to physiotherapists, nurses were older, shorter, had higher BMI, and longer work experience. In the whole study group, 30.2% of subjects reported NP, 17.7% reported thoracic pain (ThP) and 80.5% reported LBP. During working life, 90.9% of physiotherapists and 97.7% of nurses experienced spinal pain. Pain intensity measured with Visual Analogue Scale (VAS) was higher among nurses (mean 5.37) than among physiotherapists (mean 4.64). Nurses had a higher degree of disability caused by LBP and NP measured with ODI and NDI compared to physiotherapists. (4) Conclusions: Excessive strain of the spine associated with occupational activities has a strong impact on the intensity and frequency of spinal pain episodes. Physiotherapists and nurses mainly suffer from low back pain. Pain scores measured with VAS are higher in nurses than in physiotherapists.

## 1. Introduction

Neck pain (NP) and low back pain (LBP) are common musculoskeletal disorders, one of the major causes of disability globally [1,2]. LBP is regarded globally as the second most common cause of physical disability [3].

The estimated prevalence of neck pain among health care workers during one year was from 45.8% to 54.7% [4,5,6]. Neck pain may result in shorter working hours, reduced participation in recreational activities, and sleep disorders [7].

According to the Global Burden of Disease, Injuries, and Risk Factors Study (GBD 2017), LBP was the leading cause of years lived with disability (YLDs) considering all analysed conditions. About 75% to 80% of the global population will experience at least one episode of acute LBP in their lifetime [1,8]. Most patients who develop acute LBP improve within about one month. However, many patients experience persistent low-intensity pain or recurrent episodes of LBP within one year following the previous pain episode. Recent reports have emphasized the increased prevalence of LBP in young and middle-aged people [8]. LBP is the most common musculoskeletal disorder related to occupation [9]. Globally, it is estimated that in 37% of cases, LBP is occupational in nature. Work-related LBP is estimated to cause 818,000 cases of disability each year [10]. As such, LBP is an economically important problem in industrialized countries.

Occupational diseases are reported by 40 to 60% of the working-age population in most EU countries [11]. As a rule, sick leaves are more frequent and longer among women, older workers, those exposed to harder physical work and people with low socio-economic status [11,12]. According to an Irish study, long-term absence from work is caused primarily by injuries, poor mental health, and back pain [13]. In the EU countries, musculoskeletal diseases (MSDs) are the most common occupational health problem [14]. MSDs often lead to early retirement and constitute the dominant risk factor for occupational disability, especially among women [15]. Studies have also shown that MSDs are the most common occupational health problem among hospital staff, especially nurses [16,17,18].

NP and LBP might be related to the various mechanical stresses that nurses and physiotherapists are exposed to in their daily work, especially when providing care to dependent or bedridden patients. The etiology of NP and LBP is multifactorial, and the most important risk factors include age [19,20], sex [21,22], work experience [19,23,24], overweight, sedentary lifestyle [22], psychological stress [24], manual mobilization/care of patients, and insufficient education in ergonomics [19]. Occupational factors related to NP and LBP include a fast-paced work environment, repetitive movement patterns, insufficient recovery time, weight lifting, other strenuous manual effort, awkward postures, mechanical pressure, bending, twisting, vibrations, and low temperature [2,25].

Musculoskeletal diseases are a serious problem for working nurses and physiotherapists [2,26], and LBP is the most important of them, with a prevalence of 30–60% [27]. Occupational back pain has negative consequences, including absenteeism from work, loss of optimal functionality, growing costs of treatment and care, and occupational disability [28]. The identification of risk factors for NP and LBP in physiotherapists and nurses is necessary to develop screening plans and preventive programmes.

The aim of this study was to determine the influence of medical occupation (physiotherapist and nurse) on the prevalence of spinal pain, functional status, and the degree of disability.

## 2. Materials and Methods

### 2.1. Participants

A total of 544 people (462 females (84.9%) and 82 males (15.1%)), licensed to practice as a physiotherapist (n1 = 240 (44.1%)) or nurse (n2 = 304 (55.9%)) in Poland completed an anonymous questionnaire focused on spinal pain. Data collection was carried out between January and December 2021.

### 2.2. Selection Criteria

We included subjects older than 18 years of age, with a valid licence to practice and actually working as a physiotherapist or nurse. Subjects younger than 18 years, those with a history of spine injury, history of spine surgery, spine and/or lower limb malformation, and pregnant women (due to potential pregnancy-related spinal pain) were excluded from the study.

### 2.3. Instruments

Participants were surveyed using an original questionnaire, the Neck Disability Index (NDI, Polish language version) [29] and the Revised Oswestry Disability Index (ODI, Polish language version) [30]. In the original questionnaire, respondents were expected to specify or describe the characteristics of their pain. The questions focused on the experience of spinal pain in the cervical, thoracic or lumbosacral segment, pain location and duration, persistence of symptoms, alleged cause(s), and the reasons for which this pain intensified. Participants also provided information on the nature of their work, including full time/part time work, type of dominant activities at work, the number of working hours and the number of years worked. They also declared the level of their physical activity (generally defined recreation). The age, body weight and height of the participants were recorded. Body mass index (BMI) was calculated as weight (kg) divided by height squared (m^2^).

The Visual Analogue Scale (VAS) is a 10 cm line, on which the respondent is expected to mark the severity of their pain between two endpoints representing 0 (no pain) and 10 (the most extreme pain imaginable) [21].

Pain in the cervical spine was assessed using the Neck Disability Index (NDI) questionnaire, Polish language version [29]. It consists of 10 statements related to pain intensity, personal care, lifting, reading, headaches, concentration, work, driving, sleep, and recreation.

Disability caused by pain in the lumbar spine was assessed using the Oswestry Low Back Pain Disability Scale (ODI), Polish language version [30]. It consists of 10 sections with statements related to pain intensity, personal care, lifting, walking, sitting, standing, sleeping, social life, and travelling. Each statement is scored 0 to 5 points.

In NDI and ODI questionnaires each statement is scored 0 to 5 points. The total score is presented in points (0–50) or percent (0–100%):

- 0–4 points (0–8%): no disability;
- 5–14 points (10–28%): minimal disability;
- 15–24 points (30–48%): moderate disability;
- 25–34 points (50–64%): severe disability;
- 35–50 points (70–100%): extreme suffering, crippled;
- The intensity of spinal pain was measured with the Visual Analogue Scale (VAS).

### 2.4. Procedure

The data were collected using electronic questionnaires containing an original questionnaire, the Neck Disability Index (NDI, Polish language version) [29], and the Revised Oswestry Disability Index (ODI, Polish language version) [30].

An informed consent form was signed at the beginning of the test. This minimized the possibility of coercion or undue influence, and respondents had sufficient time to consider participation. Information about the purpose and nature of the research was presented to enable a voluntary decision to participate in the study. It was explained to participants that the results of the research would be used in medical studies, and they were asked to answer honestly. An online questionnaire was sent to all facilities from the Polish register of entities performing medical activities in which nurses and physiotherapists are employed. A total of 612 respondents declared their participation in the study. Of these, 31 did not meet the study inclusion criteria, and another 37 did not complete the survey correctly.

This study was approved by the Bioethics Committee at the District Medical Chambers in Gdansk (KB-14/20).

### 2.5. Statistical Analysis

All statistical calculations were performed using the data analysis software system STATISTICA version 13.3. from StatSoft Inc. (Tulsa, OK, USA, 2020), (www.statsoft.com, accessed on 1 June 2022). Quantitative variables were presented as the mean, standard deviation, median, minimum and maximum value (range) and 95% confidence interval (CI). Qualitative variables were presented as numerical values and percentages (rates). The normality of distribution of quantitative variables was verified with W Shapiro–Wilk, Lilliefors, Kolmogorov–Smirnov, and Jarque–Bera tests. The hypothesis on the *equality* of group *variances* was verified with the Brown–Forsythe test due to the different size of samples (occupation).

## 3. Results

**Figure 1:**
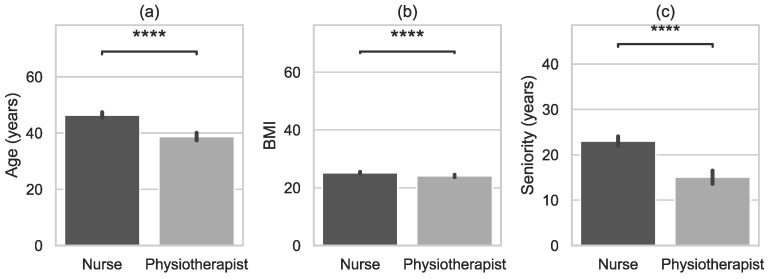
Characteristics of examined groups (occupation) with error bars and *p*-value annotation legends: (**a**) Age, (**b**) Body Mass Index (BMI), (**c**) Seniority (working years in occupation). ns: 5.00e-02 < *p* <= 1.00e + 00; *: 1.00e-02 < *p* <= 5.00e-02; **: 1.00e-03 < *p* <= 1.00e-02; ***: 1.00e-04 < *p* <= 1.00e-03; ****: *p* <= 1.00e-04.

Table 1 presents the baseline characteristics of the study population across categories of nurses and physiotherapists. Compared to physiotherapists, nurses were older, shorter, and had higher BMI (Figure 1). Work experience (seniority) in the group of nurses was longer than in the group of physiotherapists.

Table 2 presents the distribution of data on back pain at a certain level and pain radiation to one or two limbs. In the question about back pain, the respondents could select more than one answer, which means that in a given subgroup and total population the number of respondents and the rate might be higher than 100%. In both analysed subgroups, the rates were highest for low back pain and lowest for pain in the thoracic spine. Respondents from both subgroups most frequently reported central pain, located in the neck, Th spine or low back, and less frequently pain radiating to one or two limbs. Pain in the cervical spine was reported by 41.3% of physiotherapists.

Table 3 presents the characteristics of spinal pain. In both studied subgroups, back pain was experienced both by physiotherapists (90.9%) and nurses (97.7%) during their working life. Pain intensity measured with VAS was higher among nurses (mean 5.37) than among physiotherapists (mean 4.64); *p* = 0.0001 (Table 3). In both analysed subgroups the first episode of spinal pain was experienced 4 to 9 years before the study. A significant proportion of physiotherapists reported that they experienced the first pain episode one year before the study.

Most physiotherapists reported between one and five pain episodes (47.8%). The number of pain episodes most frequently reported by nurses was in the range of 1 to 5 (38.5%) or 6 to 10 (33.2%). Most physiotherapists declared they experienced back pain several times in a lifetime (35%), while most nurses reported they had pain once a day (56.6%).

For most physiotherapists, back pain caused no limitation (44.2%) or minimal limitation of physical activity (43.3%). Among nurses, spinal pain caused minimal limitation (45.1%) or significant limitation of activity at work (39.8%).

Table 4 presents specific activities that triggered pain in both analysed subgroups. In the question about back pain, the respondents could select more than one answer, which means that in a given subgroup or population the number of respondents and the rate could be higher than 100%. Physiotherapists declared that lifting was the main activity triggering pain (59.2%). Slightly lower rates were reported for standing (37.5%), bending (32.9%), and sitting (35.0%). Among nurses, the main activities triggering pain were lifting (58.6%) and bending (54.3%), followed by sitting (26.6%) and standing (26.0%).

**Figure 2:**
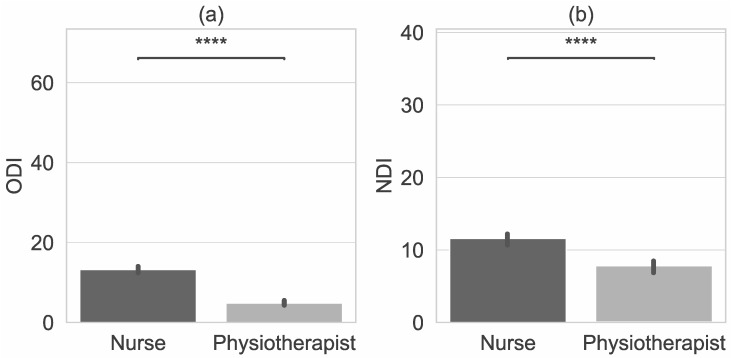
Characteristics of Physiotherapists and Nurses with error bars and *p*-value annotation legends (**a**) Oswestry Disability Index (ODI) (**b**) Neck Disability Index (NDI) values. ****: *p* <= 1.00e-04.

**Figure 3:**
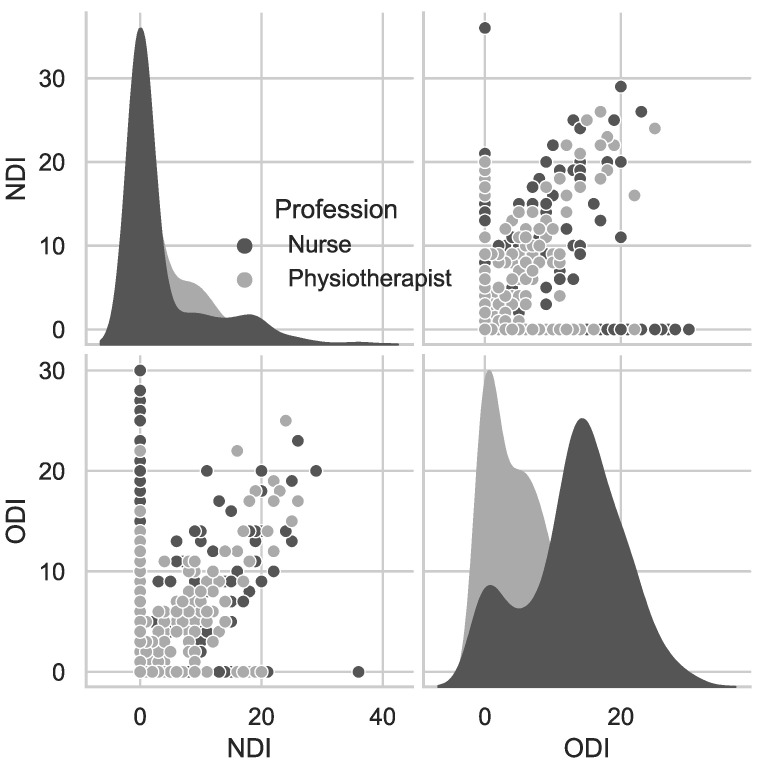
Pairwise relationship between NDI and ODI.

Table 5 shows data on the degree of disability caused by low back pain measured with ODI and pain in the cervical spine measured with NDI. The table presents only data on participants who reported low back pain and/or neck pain and completed ODI or NDI questionnaires. There was a significant difference in the degree of disability between the subgroups (*p* = 0.0001; U-M-W test). The degree of disability caused by LBP was higher in nurses than in physiotherapists (Figure 2 and Figure 3).

ODI scores indicated no disability (53.3%) or minimal disability (41.7%) in physiotherapists, and minimal disability (38.5%) or moderate disability (40.8%) in nurses (Figure 2 and Figure 3).

The degree of disability caused by neck pain was higher in nurses than in physiotherapists, despite the fact that neck pain was reported by a greater number of physiotherapists. NDI scores indicated no disability (35%) or minimal disability (55%) in physiotherapists, and minimal disability (47%) or moderate disability (34%) in nurses (Figure 2 and Figure 3).

As shown in Table 6, there was a significant relationship between pain and age and work experience (seniority). BMI had no effect on pain, despite the fact that the mean BMI (Table 1) was significantly higher in nurses that in physiotherapists.

As shown in Table 7, VAS score in nurses reporting pain was higher than in physiotherapists. 

## 4. Discussion

Spinal pain, mainly LBP, is recognized in developed countries as a frequent cause of morbidity in various occupational sectors, especially in health care workers, physicians, nurses, physiotherapists, paramedics, and midwives [31]. The incidence of back pain is likely to increase as patients become heavier and develop obesity. Therefore, more efforts are needed to predict these problems by regular assessment of physical factors associated with spinal pain in its early stages. Our study revealed that the majority of physiotherapists and nurses had spinal pain and related limitations.

In the analyzed population nurses were older, shorter, and had higher BMI compared to physiotherapists. BMI had no effect on pain intensity measured with VAS, although nurses had a higher BMI than physiotherapists. However, findings from previous studies by other authors are inconclusive. Croft et al. reported that higher body weight was a predictor of lumbosacral pain in women [32]. In a study on 3159 nurses, Chiou et al. found that low back pain was associated with lifting heavy objects, workload, age, BMI, and work habits [33]. Contrary to these observations, other researchers [34] did not show a significant association between overweight or obesity and low back pain in nurses.

In our study, age and years of work experience correlated with the intensity of back pain in both nurses and physiotherapists. Similar observations were also reported by other researchers [2]. However, Mannion et al. came to different conclusions in their prospective study. They reported that the frequency of non-specific recurrent LBP in nurses decreased along with work experience. They also suggested that this may be related to the development of protective adaptation by medical personnel to increased workload [35]. Similar results were observed in the work of podiatrists, where back problems occurred in the younger age group [36].

In our study, spinal pain measured with VAS was more severe in nurses (mean 5.37) than in physiotherapists (mean 4.64). The work experience in nurses was longer than in physiotherapists, which results from the older age of nurses. The prevalence of LBP in nurses has traditionally been attributed to high physical stress at work, such as moving patients and lifting heavy loads [37,38]. During work, nurses bend and twist when providing care to patients [39,40], and insufficiently often use the necessary aids to prevent musculoskeletal injury when handling bedridden patients. This may be due to a lack of adequate education in occupational ergonomics, lack of time, work culture or limited availability of appropriate equipment that can facilitate patient care, such as sliding boards, repositioning devices and mechanical lifts, which can reduce the risk of damage to the spine and the musculoskeletal system. Prevention of LBP primarily relies on the adherence to the principles of ergonomics, appropriate work organization and specific information given by the employer about potential risks. 

In our study, the prevalence of spinal pain in physiotherapists and nurses was comparable. In physiotherapists, however, pain measured with NDI and ODI was of lower intensity and caused lower degrees of disability. This may result from the fact that physiotherapists have a better knowledge of the structure and functioning of the musculoskeletal system and methods for the prevention of pain related to the musculoskeletal system. Nevertheless, it is alarming that spinal pain is experienced by a very high rate of physiotherapists (90.9%) and nurses (97.7%) during their working life. Physiotherapists declared they experienced back pain several times in a lifetime (35%), while nurses reported they had pain once a day (56.6%). For most physiotherapists spinal pain caused no limitation (44.2%) or minimal limitation of physical activity (43.3%). Among nurses, spinal pain caused minimal limitation (45.1%) or significant limitation of activity at work (39.8%).

In both analysed subgroups the rates were highest for low back pain (80.5%) and lowest for pain in the thoracic spine (17.7%). Respondents from both subgroups most frequently reported central pain, located in the neck (15.1%), Th spine (13.6%) and low back (46.9%), and less frequently pain radiating to one or two limbs. A study by Glowinski (Poland) revealed that 91.7% of physiotherapists experienced pain in the locomotor system during their working life, including 82% for LBP and 67% for NP [2]. However, the intensity of pain was higher in the cervical spine. Similar findings were made in the present study, where physiotherapists had NP (41.3%), Th Pain (31.7%) and LBP (70.4%), while nurses had NP (21.1%), Th Pain (6.6%) and LBP (88.5%). A systematic review by Ellapen and Narsigan showed that MSDs in nurses more frequently affected the low back, neck and shoulders [39]. According to the literature, the prevalence of LBP in nurses ranges from 43% to 77.1% [19,33,41,42]. In studies by Sikiru (Africa) [43] and Freimann (Estonia) [44], 70.87% and 57% of nurses, respectively, reported suffering from low back pain in the previous 12 months. In a study by Skela-Savič (Slovenia) the prevalence of LBP in nurses was 85.9% [45]. Physiotherapists declared that they experienced the first spinal pain episodes in school years [46]. Ellis et al. [45] reported that 27% of last-year physiotherapy students complained of spinal pain after providing rehabilitation to patients. Other researchers found that complaints of spinal pain occur within the first four or five years of working life [47,48]. While the lifetime prevalence of back pain in physiotherapists ranged between 57% and 73% [49,50], the prevalence of back pain in one year was 45% [51], and the lifetime exposure to any type of injury was 90% [47]. Rahimi et al. [52] reported that the prevalence of musculoskeletal disorders was 94% in Iranian physiotherapists. Lumbar (65%), neck (57.4%), shoulder (50.2%), upper back (49%), and knee (45.5%) were the most prevalent regions of these disorders.

A systematic review by Kuijer et al. [53] focused on the work-related risk factors for spinal pain revealed a significant relationship between lumbosacral radiculopathy and manual work, torso bending/twisting, lifting, and moving objects that involved torso bending/twisting. The research concluded that lumbosacral radiculopathy can be regarded as an occupational disease. Coenen et al. [54] presented similar findings regarding the association of low back pain with lifting and carrying. In our study, lifting (59.2%), standing (37.5%), bending (32.9%) and sitting (35.0%) were indicated by physiotherapists as the main activities triggering pain. Nurses, on the other hand, indicated that back pain was mainly caused by lifting (58.6%), bending (54.3%), sitting (26.6%) and standing (26.0%).

Our study demonstrated that the degree of disability caused by LBP was higher in nurses than in physiotherapists. ODI scores indicated no disability (53.3%) or minimal disability (41.7%) in physiotherapists, and minimal disability (38.5%) or moderate disability (40.8%) in nurses. The degree of disability caused by neck pain was higher in nurses than in physiotherapists, despite the fact that neck pain was reported by a greater number of physiotherapists than nurses. NDI scores indicated no disability (35%) or minimal disability (55%) in physiotherapists, and minimal disability (47%) or moderate disability (34%) in nurses. A study by Mroczek et al. [55] demonstrated that among all healthcare workers, nurses had the lowest scores in the quality of life, and this was related to the experienced spinal pain. The authors suggested that this may indicate the excessive effort associated with occupational activities. Similarly, other researchers have indicated that nurses are an occupational group whose work involves forced posture causing pathological spine damage, pain and disability [56,57,58]. A report by Fidecki et al. concerning nurses and paramedics working in neurology, neurosurgery, orthopaedics and traumatology departments showed that the perceived severe pain and the second and third degree of disability according to Fairbank were closely related to the workplace and length of work experience [56]. Similar findings have been reported by Maciuk et al. in nurses and Nowotny et al. in physiotherapists and midwives [59,60].

The strengths of this article are the presentation of the importance of the influence of the medical profession (physiotherapist and nurse) on the occurrence of back pain, its functional state and the degree of disability caused by pain ailments. It is important for introducing proper spine pain prevention in the physiotherapist and nursing professions. The significant difference in age and seniority between groups is the limitation of this study. Data analysing the difference by matching the age and seniority in the two groups can help to explain the greater burden in nurses.

## 5. Conclusions

The presented study indicates that actively working physiotherapists and nurses suffer from work-related pain in different segments of the spine. Excessive strain on the spine associated with occupational activities has a strong impact on the intensity of pain.

Occupational ergonomics should be introduced into the curricula of postgraduate studies and should take into account the specific nature of physiotherapist or nurse occupations. Healthcare workers should be considered in programmes for the prevention of musculoskeletal pain and should have an opportunity to attend regular courses in occupational ergonomics. Healthcare facilities should be equipped with ergonomic aids.

Spinal pain ailments among physiotherapists and nurses most often concern LBP.

Pain scores measured with VAS are higher in nurses than in physiotherapists.

The degree of disability caused by neck pain (measured with NDI) and low back pain (measured with ODI) is higher in nurses than in physiotherapists.

## Figures and Tables

**Table 1:** Baseline characteristics of the study population across categories of nurses and physiotherapists.

KERRYPNX		Study Population, Total (N = 544)	Physiotherapists (n1 = 240)	Nurses (n2 = 304)	*p*-Value
Sex	Number (% of study population)	Women 462 (84.9%) Men 82 (15.1%)	Women 169 (70.4%) Men 71 (29.6%)	Women 293 (95.8%) Men 11 (4.2%)	
Age (categories)	Number (% of study population)				
20–29		56 (10.3%)	39 (16.3%)	17 (5.6%)	
30–39		161 (29.6%)	109 (45.4%)	52 (17.1%)	
40–49		130 (23.9%)	33 (13.8%)	97 (31.9%)	
≥50		197 (36.2%)	59 (24.6%)	138 (45.4%)	
Age (years)	Mean (SD)	43.0 (10.6)	38.7 (11.0)	46.4 (9.0)	0.0001 ^1^
	Range	19–65	19–63	24–65	
	Me	43	35	48	
	(95% CI)	(42.1; 43.9)	(37.3; 40.1)	(45.4; 47.4)	
Height (cm)	Mean (SD)	168.4 (7.7)	171.0 (8.6)	166.4 (6.2)	0.0001 ^1^
	Range	150–199	153–199	150–198	
	Me	168	170	166	
	(95% CI)	(167.7; 169.0)	(169.9; 172.1)	(165.7; 167.0)	
Weight (kg)	Mean (SD)	70.2 (10.9)	70.9 (13.9)	69.6 (7.7)	0.4054 ^1^
	Range	42–120	42–120	48–102	
	Me	69	67	70	
	(95% CI)	(69.2; 71.1)	(69.1; 72.6)	(68.7; 70.5)	
BMI	Mean (SD)	24.7 (3.1)	24.1 (3.4)	25.2 (2.8)	0.0001 ^1^
	Range	17.0–37.9	17.0–33.9	18.0–37.9	
	Me	24.7	23.5	25.0	
	(95% CI)	(24.4; 25.0)	(23.7; 24.5)	(24.9; 25.5)	
Seniority (years)	Mean (SD)	19.5 (11.3)	15.1 (11.8)	23.0 (9.6)	0.0001 ^1^
	Range	1–42	1–42	1–41	
	Me	20	12	25	
	(95% CI)	(18.6; 20.5)	(13.6; 16.6)	(21.9; 24.1)	

^1^ U-M-W test.

**Table 2:** Back pain prevalence rates based on occupation.

	Back Pain at A Certain Level n (%)							
	Neck Pain			Th Spine Pain		Low Back Pain		
Total population	164 (30.2%)			96 (17.7%)		438 (80.5%)		
Physiotherapists	99 (41.3%)			76 (31.7%)		169 (70.4%)		
Nurses	64 (21.1%)			20 (6.6%)		269 (88.5%)		
	Central	To 1 limb	To 2 limbs	Central	Radiation	Central	To 1 limb	To 2 limbs
Total population	82 (15.1%)	57 (10.5%)	22 (4.0%)	74 (13.6%)	29 (5.3%)	255 (46.9%)	185 (34.0%)	21 (3.9%)
Physiotherapists	67 (27.5%)	36 (14.8%)	10 (4.1%)	56 (23.0%)	29 (11.9%)	109 (44.7%)	80 (32.8%)	5 (2.1%)
Nurses	15 (4.9%)	21 (6.9%)	12 (7.5%)	18 (6.9%)	0 (0.0%)	146 (48.0%)	105 (34.5%)	16 (5.3%)

C spine—cervical spine; Th spine—thoracic spine; L-S spine—lumbosacral spine; Central—pain radiation centrally; To 1 limb—Pain radiation to 1 limb; To 2 limbs—pain radiation to 2 limbs.

**Table 3:** Characteristics of pain reported by nurses and physiotherapists.

		Total Population (N = 544)	Physiotherapists (n1 = 240)	Nurses (n2 = 304)	*p*-Value
Spinal pain experienced during working life	Yes	517 (95.0%)	220 (90.9%)	297 (97.7%)	
	No	27 (5.0%)	20 (9.1%)	7 (2.3%)	
Intensity of pain episodes (VAS)	Mean (SD)	4.94 (1.6)	4.64 (1.6%)	5.37 (1.3)	0.0000 ^1^
	Range	0–10	0–10	2–8	
	Me	5	5	5	
	(95% CI)	(4.8; 5.1)	(4.4; 4.8)	(5.2; 5.6)	
First episode of spinal pain (years)	1 year ago	69 (13.1%)	52 (23.2%)	17 (5.6%)	
	2–3 years ago	91 (17.2%)	31 (13.8%)	60 (19.7%)	
	4–6 years ago	157 (29.7%)	53 (23.7%)	104 (34.3%)	0.0000 ^1^
	7–9 years ago	130 (24.6%)	56 (25.0%)	74 (24.3%)	
	≥10 years ago	77 (14.6%)	32 (14.3%)	45 (14.8%)	
	missing data	4 (0.8%)	0 (0%)	4 (1.3%)	
Number of experienced episodes of spinal pain (number)	0	38 (7%)	22 (9.2%)	16 (5.3%)	
	1–5	231 (42.7%)	115 (47.8%)	116 (38.5%)	
	6–10	147 (27.2%)	46 (19.2%)	101 (33.2%)	0.0045 ^1^
	≥11	125 (23.1%)	57 (23.8%)	68 (22.4%)	
	missing data	3 (0.6%)	0	3 (1.0%)	
Frequency of pain episodes	no pain	21 (3.9%)	16 (6.7%)	5 (1.6%)	
	once in a lifetime	8 (1.5%)	6 (2.5%)	2 (0.7%)	
	several times in a lifetime	101 (18.6%)	84 (35.0%)	17 (5.6%)	
	once a year	21 (3.9%)	19 (7.9%)	2 (0.7%)	0.0000 ^1^
	once a month	63 (11.6%)	46 (19.2%)	17 (5.6%)	
	once a week	78 (14.3%)	25 (10.4%)	53 (17.4%)	
	once a day	192 (35.3%)	20 (8.3%)	172 (56.6%)	
	all the time	60 (11.0%)	24 (10.0%)	36 (11.8%)	
Limitation of physical activity	no	151 (27.8%)	106 (44.2%)	45 (14.8%)	
	minimal	243 (44.7%)	106 (43.4%)	137 (45.1%)	
	significant	147 (27.0%)	26 (10.7%)	121 (39.8%)	0.0058 ^1^
	disabling	3 (0.6%)	2 (0.9%)	1 (0.3%)	

VAS—Visual Analogue Scale; ^1^—Chi-square.

**Table 4:** Activities triggering pain.

	Total Population (N = 544)	Physiotherapists (n1 = 240)	Nurses (n2 = 304)
Lifting	320 (58.8%)	142 (59.2%)	178 (58.6%)
Bending	244 (44.9%)	79 (32.9%)	165 (54.3%)
Standing	169 (31.1%)	90 (37.5%)	79 (26.0%)
Sitting	165 (30.3%)	84 (35.0%)	81 (26.6%)
Torso twist	84 (145.4%)	52 (21.7%)	32 (10.5%)
Torso hyperextension	35 (6.4%)	21 (8.8%)	14 (4.6%)

**Table 5:** ODI and NDI scores in physiotherapists and nurses.

		Total Population	Physiotherapists	Nurses
ODI				
Number of participants with LBP who completed the ODI questionnaire		438	169	269
ODI	Mean (SD)	10.4 (7.3)	6.0 (4.9)	13.2 (7.2)
	Range	0–30	0–25	0–30
	Me	10	5	14
	(95% CI)	(9.8; 11.0)	(5.3; 6.7)	(12.4; 14.0)
0–4 points (0–8%) no disability	Number (% of total population, or any of the subgroup)	no 177 (32.5%)	no 128 (53.3%)	no 49 (16.1%)
5–14 points (10–28%) minimal disability		minimal 216 (39.7%)	minimal 100 (41.7%)	minimal 116 (38.5%)
15–24 points (30–48%) moderate disability		moderate 135 (24.9%)	moderate 11 (4.6%)	moderate 124 (40.8%)
25–34 points (50–64%) severe disability		severe 16 (2.9%)	severe 1 (0.4%)	severe 15 (4.9%)
35–50 points (70–100%) crippled		crippled 0 (0%)	crippled 0 (0%)	crippled 0 (0%)
NDI				
Number of participants with NP who completed the NDI questionnaire		163	99	64
NDI	Mean (SD)	9.6 (7.0)	8.0 (6.3)	12.3 (7.3)
	Range	1–36	1–26	1–36
	Me	8	7	12
	(95% CI)	(9.0; 10.2)	(7.3; 8.7)	(11.5; 8.1)
0–4 points (0–8%) no disability	Number (% of total population, or any of the subgroup)	no 43 (26%)	no 35 (35%)	no 8 (13%)
5–14 points (10–28%) minimal disability		minimal 84 (52%)	minimal 54 (55%)	minimal 30 (47%)
15–24 points (30–48%) moderate disability		moderate 31 (19%)	moderate 9 (9%)	moderate 22 (34%)
25–34 points (50–64%) severe disability		severe 4 (2%)	severe 1 (1%)	severe 3 (5%)
35–50 points (70–100%) crippled		crippled 1 (1%)	crippled 0 (0%)	crippled 1 (1%)

ODI—Oswestry Low Back Pain Disability Scale; NDI—Neck Disability Index; LBP—Low Back Pain; NP—Neck Pain.

**Table 6:** Results.

		Pain		*p*-Value
		Yes	No	
Age (years)	Total population	43.0 (10.6)	32.1 (8.3)	0.0001 ^1^
	Physiotherapists Nurses	39.3 (10.9) 46.7 (8.7)	32.4 (9.1) 31.6 (5.7)	0.0021 ^1^ 0.0002 ^1^
BMI	Total population	24.8 (3.1)	23.4 (3.3)	0.0920 ^1^
	Physiotherapists Nurses	24.2 (3.4) 25.2 (2.8)	23.1 (3.5) 24.1 (2.8)	0.3210 ^1^ 0.4209 ^1^
Seniority (years)	Total population Physiotherapists Nurses	20.1 (11.1) 15.6 (11.8) 23.4 (9.3)	8.8 (8.9) 9.8 (9.8) 6.0 (5.5)	0.0001 ^1^ 0.0152 ^1^ 0.0001 ^1^

^1^ U-M-W test.

**Table 7:** VAS scores in physiotherapists and nurses.

		Total Population (N = 544)	Physiotherapists (n1 = 240)	Nurses (n2 = 304)	*p*-Value
Pain	Yes	5.0 (1.5)	4.7 (1.6)	5.3 (1.3)	0.0001 ^1^

^1^ U-M-W test.

## Data Availability

The datasets used and/or analyzed during the present study are available from the corresponding author.
